# Successful use of closed-loop allostatic neurotechnology for post-traumatic stress symptoms in military personnel: self-reported and autonomic improvements

**DOI:** 10.1186/s40779-017-0147-0

**Published:** 2017-12-22

**Authors:** Catherine L. Tegeler, Lee Gerdes, Hossam A. Shaltout, Jared F. Cook, Sean L. Simpson, Sung W. Lee, Charles H. Tegeler

**Affiliations:** 1 Department of Neurology, Wake Forest School of Medicine, Medical Center Boulevard, Winston-Salem, NC 27157 USA; 2 Brain State Technologies, LLC, 15150 North Hayden Road, Scottsdale, AZ 85260 USA; 3 Hypertension and Vascular Research Center, Wake Forest School of Medicine, Medical Center Boulevard, Winston-Salem, NC 27157 USA; 4 Department of Biostatistical Sciences, Wake Forest School of Medicine, Medical Center Boulevard, Winston-Salem, NC 27157 USA

**Keywords:** Neurotechnology, Allostasis, Autonomic, Heart rate variability, Baroreflex sensitivity, Closed-loop, Acoustic stimulation, Military, Post-traumatic stress disorder, HIRREM

## Abstract

**Background:**

Military-related post-traumatic stress (PTS) is associated with numerous symptom clusters and diminished autonomic cardiovascular regulation. High-resolution, relational, resonance-based, electroencephalic mirroring (HIRREM®) is a noninvasive, closed-loop, allostatic, acoustic stimulation neurotechnology that produces real-time translation of dominant brain frequencies into audible tones of variable pitch and timing to support the auto-calibration of neural oscillations. We report clinical, autonomic, and functional effects after the use of HIRREM® for symptoms of military-related PTS.

**Methods:**

Eighteen service members or recent veterans (15 active-duty, 3 veterans, most from special operations, 1 female), with a mean age of 40.9 (SD = 6.9) years and symptoms of PTS lasting from 1 to 25 years, undertook 19.5 (SD = 1.1) sessions over 12 days. Inventories for symptoms of PTS (Posttraumatic Stress Disorder Checklist – Military version, PCL-M), insomnia (Insomnia Severity Index, ISI), depression (Center for Epidemiologic Studies Depression Scale, CES-D), and anxiety (Generalized Anxiety Disorder 7-item scale, GAD-7) were collected before (Visit 1, V1), immediately after (Visit 2, V2), and at 1 month (Visit 3, V3), 3 (Visit 4, V4), and 6 (Visit 5, V5) months after intervention completion. Other measures only taken at V1 and V2 included blood pressure and heart rate recordings to analyze heart rate variability (HRV) and baroreflex sensitivity (BRS), functional performance (reaction and grip strength) testing, blood and saliva for biomarkers of stress and inflammation, and blood for epigenetic testing. Paired *t*-tests, Wilcoxon signed-rank tests, and a repeated-measures ANOVA were performed.

**Results:**

Clinically relevant, significant reductions in all symptom scores were observed at V2, with durability through V5. There were significant improvements in multiple measures of HRV and BRS [Standard deviation of the normal beat to normal beat interval (SDNN), root mean square of the successive differences (rMSSD), high frequency (HF), low frequency (LF), and total power, HF alpha, sequence all, and systolic, diastolic and mean arterial pressure] as well as reaction testing. Trends were seen for improved grip strength and a reduction in C-Reactive Protein (CRP), Angiotensin II to Angiotensin 1–7 ratio and Interleukin-10, with no change in DNA n-methylation. There were no dropouts or adverse events reported.

**Conclusions:**

Service members or veterans showed reductions in symptomatology of PTS, insomnia, depressive mood, and anxiety that were durable through 6 months after the use of a closed-loop allostatic neurotechnology for the auto-calibration of neural oscillations. This study is the first to report increased HRV or BRS after the use of an intervention for service members or veterans with PTS. Ongoing investigations are strongly warranted.

**Trial registration:**

NCT03230890, retrospectively registered July 25, 2017.

## Background

Advanced understanding and treatment for post-traumatic stress disorder (PTSD) will require a paradigm that appreciates its complexity and holds a promising solution for its extensive burden of suffering. Conventionally, PTSD is classified and treated as a behavioral disturbance that can follow a traumatic event, and its main symptom clusters pertain to re-experiencing the trauma, avoidance and generalized numbing, negative cognitions and mood, and heightened arousal [1]. However, while military service members with PTSD are beset with increased psychosocial risks, including compromised role functions [2], substance abuse [3], and suicidality [4], studies also show an increased risk for cardiovascular and metabolic diseases [5, 6] as well as all-cause mortality [7]. Care for individuals with traumatic stress symptomatology should thus entail attention to both behavioral and physical health status. Moreover, although therapies based on re-exposure to trauma have been designated as evidence-based treatments [8], there is concern about high dropout rates associated with this approach [9], as well as a lack of impact on sleep disturbances [10]. In addition, despite the undeniable suffering of American veterans of the Vietnam War, which contributed to recognition of PTSD as a Diagnostic and Statistical Manual of Mental Disorders (DSM) clinical disorder, there are also concerns that the medicalization of combat-related stress can lead to unhelpful consequences related to stigmatization [11, 12].

To improve modeling of PTSD (or post-traumatic stress, PTS, more broadly), a promising starting point may be the recognition of the brain as the organ of central command. The physiological paradigm of *allostasis* (stability through change) notes that the brain directs set points for biological function on the basis of perceived needs for organism-level survival [13, 14]. Stated another way, it is the brain that lets environmental stressors “get under the skin” [15]. Within the allostasis paradigm, the sympathetic and parasympathetic divisions of the autonomic nervous system serve as principal pathways for bidirectional communication and coordination between the brain and peripheral physiology. For example, in the setting of an acute threat, the brain orchestrates these divisions to affect an instantaneous redirection of metabolic resources away from the anabolic process of digestion toward the catabolic process of mobilization. Allostasis thus fully predicts that over time, exposure to traumatic stresses is likely to entail both behavioral and physical health disturbances. For therapeutics, allostasis points to the potential for multi-system symptom reduction through interventions that are expressly designed to facilitate the brain’s role as the organ of central command [16]. Moreover, through its emphasis on context-dependent heightened stress responsivity, the allostasis paradigm supports destigmatization of PTS-related phenomenology. There is no single normal mode of brain function; rather, there are ranges for set points [17] that are subject to modification based on neuroplasticity. The functional set point for a given capacity—vigilance, for example—may or may not be adaptive (“pathological”), depending on the environment.

Given its crucial and distributed role for connecting the brain, body, and behavior, the autonomic nervous system merits special attention in the allostasis paradigm. Autonomic regulation of the cardiovascular system can be characterized by measuring heart rate variability (HRV) and baroreflex sensitivity (BRS). HRV and BRS indicate the physiological capacity to produce dynamically varied responses to the changing needs of an environment, and prospective studies show that decreased HRV is a risk factor for incident cardiovascular disease [18] and all-cause mortality [19]. Furthermore, depressed HRV is seen generally across behavioral disorders [20], specifically in military personnel and veterans with diagnosed PTSD [21–25], and as a pre-deployment predictor of new post-deployment PTSD diagnoses or symptom severity [26, 27].

An open question in studies of PTS and autonomic dysregulation pertains to how or why HRV is depressed in persons who have been exposed to traumatic stress. An epigenetic and neural oscillatory explanation is provided by the bihemispheric autonomic model (BHAM) [28]. The BHAM begins by recognizing that there is hemispheric lateralization in the management of the autonomic nervous system, with the right and left sides having primary responsibility for the sympathetic and parasympathetic divisions, respectively. The BHAM proposes that trauma-related sympathetic hyperarousal may be an expression of maladaptive right temporal lobe activity, whereas the avoidant and dissociative features of the traumatic stress response may be indicators of a parasympathetic “freeze” response that is significantly driven by the left temporal lobe. An implication of the BHAM is that a successful allostatic (i.e., brain-based, top-down) intervention may facilitate the reduction of symptom clusters associated with autonomic disturbances through the mitigation of maladaptive asymmetries.

The objective of this report is to document changes in self-reported symptoms, autonomic, and functional measures after use of a closed-loop acoustic stimulation neurotechnology by a series of active duty service members or recent veterans with military-related symptoms of traumatic stress. The neurotechnology strategy (HIRREM®; Brain State Technologies, Scottsdale, Arizona) is aligned with the allostasis paradigm through its brain-focused strategy for the auto-calibration of neural oscillations [16]; through its attention to hemispheric asymmetry, it is also designed to leverage insights described in the BHAM. We hypothesized that use of the neurotechnology would be followed by reductions in self-reported PTSD-related symptomatology as well as improvements in HRV and BRS. For a subset of the initial subjects, we also conducted exploratory analysis of changes in biochemical and epigenetic markers related to stress or inflammation. Changes in brain network connectivity demonstrated through analysis of whole brain, resting-state magnetic resonance imaging (MRI) evaluations are reported elsewhere [29, 30].

## Methods

### Population and subject recruitment

This single site, ongoing, IRB-approved pilot study (Clinicaltrials.gov registration NCT0323089), is being carried out in the Department of Neurology at the Wake Forest School of Medicine, Winston-Salem, North Carolina, USA. Initial eligibility screening is conducted through an online questionnaire followed by a phone conversation. To be considered for inclusion, individuals must be active duty military service members or recent veterans with service since 2001 with symptoms of military-related traumatic stress, including insomnia, poor concentration, sadness, irritability, or hyper-alertness, with or without a history of traumatic brain injury (TBI). Participants are required to have either a formal diagnosis of PTSD, a referral from a military medical provider confirming active PTS symptoms, or prior or current treatment for the same. For those participants who are special operations service members, the study deliberately does not use a symptom inventory threshold score as an eligibility criterion because of the under-reporting of symptoms among these individuals (personal communication, Naval Special Warfare medical officer). If contact is established through self-referral with the absence of a formal PTSD diagnosis, a score of 50 points on a screening PCL-M is required. Potential participants have been identified by referrals from military medical providers, as well as the Care Coalition and Preservation of the Force and Family, which both support the special operations community of the United States Armed Forces. Several participants have joined through self-referral after word of mouth from other participants or review of open studies on the research program webpage on the Wake Forest Baptist Health website. Based on advice from military personnel and recognition that the potential stigma associated with a diagnosis of PTSD might limit recruitment, study flyers and related materials focused on symptoms and did not include use of the term “PTSD.”

Exclusion criteria are the inability to provide informed consent, inability to attend all study visits or sit comfortably in a chair, bilateral total hearing loss, known seizure disorder, or an ongoing need for the use of benzodiazepines, opiates, anti-psychotic medications, selective serotonin reuptake inhibitor (SSRIs) or selective norepinephrine reuptake inhibitor (SNRIs), prescribed sleep medications including zolpidem or eszopiclone, stimulant medication, or thyroid hormones. Those with ongoing or anticipated regular use of recreational drugs, alcohol, or energy drinks during the intervention and in the 4 weeks following intervention completion or a lack of internet or smart phone access were also excluded. With the knowledge of and under the direct management of their medical provider, participants could titrate off what would otherwise have been considered exclusionary medications or recreational substances prior to enrollment.

### Intervention schedule

Beginning on a Monday morning, and following informed consent, baseline (Visit 1, V1) outcome measures are collected (details below), including self-reported symptom inventories, physiological and functional measures, an assessment of brain electrical activity, blood and saliva samples for biomarkers or epigenetic testing, and a whole brain, resting-state MRI scan. Participants then receive a series of closed-loop acoustic stimulation sessions (HIRREM) over a period of 12 days. The initial two sessions are given on the afternoon of the first day following the completion of all baseline data collections. Thereafter, participants receive two sessions daily, with a break between sessions. Typically, no sessions are given on Saturday (day 6), many receive a single afternoon session on Sunday (day 7), with a final, single morning session on the second Friday (day 12), prior to the repeated outcome measures.

All outcome measures are repeated prior to departure on day 12 (Visit 2, V2), except that for scheduling purposes and to ensure similar time of day of sampling, blood and saliva collection followed the morning session on day 11. Symptom inventories are collected remotely via online surveys at 1, 3, and 6 months following intervention completion (V3, V4, V5, respectively). Brief informal interviews are conducted with participants in person during V2 data collection, and narrative comments are sought at subsequent data collections by either phone or email.

### Outcome measures

#### Symptom inventories

A panel of outcome measures evaluate clinical symptoms related to PTSD, insomnia, depressive mood, and anxiety. The Posttraumatic Stress Disorder Checklist, military version (PCL-M) measures the American Psychiatric Association’s Diagnostic and Statistical Manual of Mental Disorders (DSM-IV) Criteria B, C, and D for PTSD symptoms based on traumatic life experiences related to military service [31]. Seventeen items are rated on a Likert scale with a composite score range of 17 to 85. A score of 50 or higher is correlated with a probability of military-related PTSD [32], although cutoff scores as low as 30 to 34 have been suggested for active-duty soldiers seen in primary care populations [33]. A reduction of ≥ 10 points in the PCL-M has been suggested to be a clinically significant change [34]. The Insomnia Severity Index [35] is a 7-question measure, with responses from 0 to 4 for each question, that yields scores ranging from 0 to 28. A score of 15 or greater is considered to indicate moderate or greater insomnia severity, and 8 to 14 indicates subthreshold insomnia. A reduction of at least 6 to 7 points has been suggested as the minimally important clinical difference for insomnia symptom reduction [36, 37]. The Center for Epidemiologic Studies Depression Scale (CES-D) [38] is a 20-item survey assessing affective depressive symptomatology to screen for the risk of depression. Scores range from 0 to 60, and a score of 16 or greater is commonly used as a clinically relevant cut-off [39]. The Generalized Anxiety Disorder 7-item scale (GAD-7) [40] is a seven-item screening tool for anxiety that is widely used in primary care. The clinical threshold to consider treatment is 8, and a statistically reliable change is 4 or greater. Subjects with a history of mild traumatic brain injury or concussion also complete the Rivermead Post-Concussion Questionnaire [41], a 16-item survey that assesses the severity of common post-concussion symptoms on a scale of 0 to 4, with a total score range from 0 to 64 (least to highest symptom severity).

#### Autonomic cardiovascular regulation

Continuous recordings of blood pressure (BP) and heart rate (HR) are obtained from noninvasive finger arterial pressure measurements and electrocardiogram for 10 min with subjects resting supine and breathing freely. These recordings follow the completion of the symptom inventories and functional testing. Systolic, diastolic, and mean arterial BP, as well as beat-to-beat RR intervals (RRI) files generated via the data acquisition system (BIOPAC acquisition system and Acknowledge 4.2 software, Santa Barbara, CA) at 1000 Hz are analyzed using Nevrokard SA-BRS software (by Nevrokard Kiauta, d.o.o., Izola, Slovenia). All recordings are visually inspected, and the first 5 min of usable tracings are analyzed. Recordings with dropped beats or gross motion artifacts are excluded from analysis. Assessments included multiple measures of heartrate variability (HRV) in both time and frequency domains, baroreflex sensitivity (BRS), and blood pressure [42].

#### Functional testing

Reaction testing uses a drop-stick, clinical reaction time apparatus. It is constructed from a meter stick covered in friction tape with gradations. The modified meter stick is fixed to a weighted rubber cylinder. The apparatus is placed between the thumb and index finger of the subject and released at a random time during a countdown. The subject catches the apparatus and the distance it has fallen is measured. Following two practice trials, subjects perform 8 trials, and the mean distance value is used for analysis [43]. Grip strength evaluation is done using a hydraulic hand dynamometer (Baseline Hydraulic Hand Dynamometer). The greatest force generated during three trials is used for analysis [44].

#### Biomarkers of stress and inflammation and epigenetic measures

During the study, funding became available to permit limited exploratory analysis of post-interventional changes in markers of stress and inflammation in 15 subjects and for epigenetic measures in 8 subjects. Blood-based measures included Angiotensin II (Ang II), Angiotensin 1 to 7 (Ang 1–7), epinephrine, norepinephrine, C-reactive protein (CRP), vasopressin, Interleukin 1 (IL-1), Interleukin 6 (IL-6), and Interleukin 10 (IL-10), and saliva measures included cortisol and alpha-amylase. For epigenetic testing, DNA was isolated from whole blood samples to quantify DNA methylation at each site. Microarray assays were used to determine the methylation proportion for each site (beta value) based on the ratio of the fluorescence intensity of the methylated versus the combined methylated and unmethylated probes.

#### Closed-loop allostatic neurotechnology intervention

The process and procedures for the provision of closed-loop allostatic neurotechnology by a technologist in an office setting have been discussed in detail previously [16]. An initial assessment of brain electrical activity entails two-channel recordings from at least 6 paired locations on the scalp (F3/F4, C3/C4, T3/T4, P3/P4, FZ/OZ, O1/O2; also, typically FP1/FP2 and CB1/CB2) with the participant at rest and while carrying out a task, using sensors and amplifiers that sample at 256 Hz. At each scalp location, data are recorded for 1 minute each with eyes closed, eyes partially open as a transitional state of arousal, and eyes open while carrying out a specific mental task (e.g., reading numbers or performing mental calculations). Trained technologists evaluate assessment data to choose protocols for the initial intervention session.

Protocols for each session include recording brain electrical activity through, generally, two channels, with scalp sensors placed at homologous regions of the hemispheres according to the 10–20 International EEG system. Software algorithms analyze specific ranges of the brain electrical frequency spectrum in real time, identify dominant frequencies based on proprietary mathematical formulae, and translate those frequencies into acoustic stimuli (audible tones of variable pitch and timing) which are presented to participants through standard earphones (Creative EP-630 or Sony Stereo Headphones MDR-EX58V) with as little as an eight-millisecond delay. Volume (decibels) of acoustic stimulation is adjusted for each participant in accordance with their preference.

Each session (typically 90–180 min each) consists of 4 to 10 protocols, ranging from 5 to 40 min per protocol, and each is intended to address a specific anatomical location and frequency range. Some protocols are completed with eyes closed and some with eyes open, with the participant being asked to relax while sitting or reclining comfortably in a zero-gravity chair. After the initial session, specific protocols and protocol durations for successive sessions are chosen based on brain electrical data from the participant’s preceding session, which, for purposes of technologist review, are aggregated in broad-band frequency ranges (< 1.0 Hz; 1.0–3.0 Hz; 3.0–5.5 Hz; 5.5–7.5 Hz; 7.5–10.0 Hz; 10.0–12.0 Hz; 12.0–15.0 Hz; 15.0–23.0 Hz; 23.0–36.0 Hz; 36.0–48.0 Hz). Special attention is given to activity set points suggestive of dominant hemispheric asymmetries and/or suboptimal ratios of energy across the frequency spectrum. Algorithms are designed to support the de-establishment of relatively invariant and potentially maladaptive activity patterns. All participants continued with their current medical or behavioral care being used at the time of enrollment.

Although exact mechanisms await confirmation, it appears that with rapid updates regarding its own electrical activity, intended to support frequency-matching or resonance between the acoustic stimulation and oscillating brain networks, the brain is supported towards auto-calibration and self-optimization. As a closed-loop process, no conscious or cognitive activity is required, yet the brain pattern is observed to shift on its own terms towards improved balance and, often, reduced hyperarousal.

#### Statistical analysis

A repeated-measures ANOVA was performed to evaluate changes in symptom inventory scores between baseline and each follow-up visit. For other comparisons, two-tailed paired t-tests were performed to evaluate pre- to post-HIRREM changes. In consideration of the sample size, the non-parametric Wilcoxon signed-rank test was used to corroborate the t-test findings. Analyses were performed using SAS (Cary, NC).

## Results

Twenty-seven individuals were screened, and 18 met eligibility criteria, provided informed consent, and enrolled in the study. Of the 9 who were excluded, 7 had schedule or training cycle conflicts that did not permit travel to the study site, and 2 did not meet criteria with respect to a formal diagnosis of PTSD, active symptoms, or treatment of PTS. The mean age of the cohort was 40.9 (SD 6.9) years. There were 17 men, and the cohort was largely Caucasian (17 Caucasian, 1 Asian). Three recent veterans were enrolled, while the other 15 participants were on active duty. Self-reported health conditions are listed in Table 1, and therapies previously used for PTS symptom remediation are listed in Table 2. Of the 11 individuals who reported previous use of a psychoactive or sleep-related medication, 10 had made recent adjustments to their regimen (withholding or discontinuing a medication that would entail exclusion) under the guidance of their medical provider. Participants received a mean of 19.5 (SD 1.1) HIRREM sessions, with 2779 min (SD 315) of protocol time, over the 12-day intervention period. There were no adverse events and no drop-outs. One participant temporarily returned to his military base midway through the intervention period to be closer to his social support network and to address some active-duty responsibilities. Nine HIRREM sessions were provided to him by one of the study investigators (CLT) at his location using a mobile configuration of the HIRREM intervention (laptop instead of desktop computer). Table 3 provides the military service history for each participant, including their duration of traumatic stress symptoms and number of recognized traumatic brain injuries (TBIs) as well as selected notes that pertain to their experience with the study during and after the intervention.

**Table 1:** Self-reported health conditions

Condition	Number of participants reporting
Traumatic brain injury or concussion	15
Insomnia	11
Impaired memory or cognitive ability	10
Depression	9
Stress or anxiety	9
Tinnitus	9
Chronic pain	7
Chronic fatigue	7
Headaches	5
Vertigo or dizziness	5
Migraines	4
Hot flashes	4
Attention deficit (hyperactivity) disorder	4
Lipid disorder	4
Fibromyalgia	1
Learning disability	1

**Table 2:** Interventions reported as previous treatments for PTS(D)

Type of intervention	Number of participants reporting
Psychotropic or sleep-related medications or supplements	11
Cognitive behavioral therapy/psychotherapy	8
Program at National Intrepid Center of Excellence	3
Eye movement desensitization and reprocessing	2
Biofeedback	2
Relaxation therapy/progressive muscle relaxation	2
Physical therapy	2
Chiropractor	2
Occupational therapy	2
Transcranial direct-current stimulation	1
Group therapy	1
Stretch therapy	1
Yoga	1
Reiki	1
Shin Jin Jitsu	1
Service dog	1
Speech therapy	1
Hearing aid	1

**Table 3:** Individual service, traumatic stress, and TBI histories and intervention-related notes or observations

Branch of US armed services	Years in military/number of deployments	Years with PTS(D) symptoms	Number of TBIs or concussions (including sport-related)	Selected intra- or post-interventional notes or observations
Navy	23/14	2	1	Able to decrease use of chewing tobacco by one-third
Navy	14/14	10	22	Easier to go for runs
Navy	26/9	12	0	“Short fuse” better, increased capacity for work-outs, decreased need for CPAP machine for sleep
Navy	30/9	n/a	15	Improved energy, less ringing in ears, “physically the best I have felt in years”
Navy	10/2	5	1	Handling stressors better, working out much more after intervention
Navy	17/8	11	3	Found it difficult to relax and sit still in session chair
Navy	33/10	10	3	9 sessions provided at his location (see text); handling stress better, more energy
Navy	26/8	4	0	“More optimistic about the future, sleep is so much better, 80% reduction in irritability”
Navy	8/2	2+	0	Tics have improved, no longer living in a constant state of panic
Navy	8/3	2.5	4	“Memory is through the roof, no longer feels like fighting cotton balls in my head”
Navy	29/2	25	0	“Teaching driving skills to son without being so controlling and angry”
Navy	28/11	7	0	Improved sleep pattern and quality
Navy	15/14	4	1	“Talking to people more, stomach is better”
Navy	19/4	1	0	“Aggression level and irritability have decreased some”
Navy	25/19	8	4	“Less worn down”
Air Force	11/8	7.5	0	Sleep was worse in the week after intervention, “workouts are good”
Navy	22/6	4	0	Little to no night sweats
Navy	13/4	6	0	“Highly recommendable, monthly use would be valuable”

n/a. data is not available; CPAP. continuous positive air pressure

**Fig. 1:**
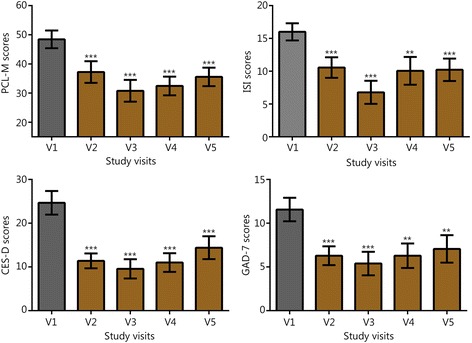
Scores for symptom inventories at the baseline study visit (V1) and at subsequent follow-up data collection visits (V2 through V5) following the use of a closed-loop allostatic neurotechnology. Symptom inventories include the PTSD Checklist − Military version (PCL-M), Insomnia Severity Index (ISI), Centers for Epidemiological Studies-Depression (CES-D), and Generalized Anxiety Disorder-7 (GAD-7). Error bars reflect the standard error of the mean. Statistical significance based on a repeated-measures ANOVA for changes between baseline and each follow-up visit is reflected as **, *P* < 0.01; ***, *P* ≤ 0.001. V1. baseline study visit; V2-V5. immediately after (V2), and at 1 (V3), 3 (V4), and 6 (V5) months after intervention completion

**Fig. 2:**
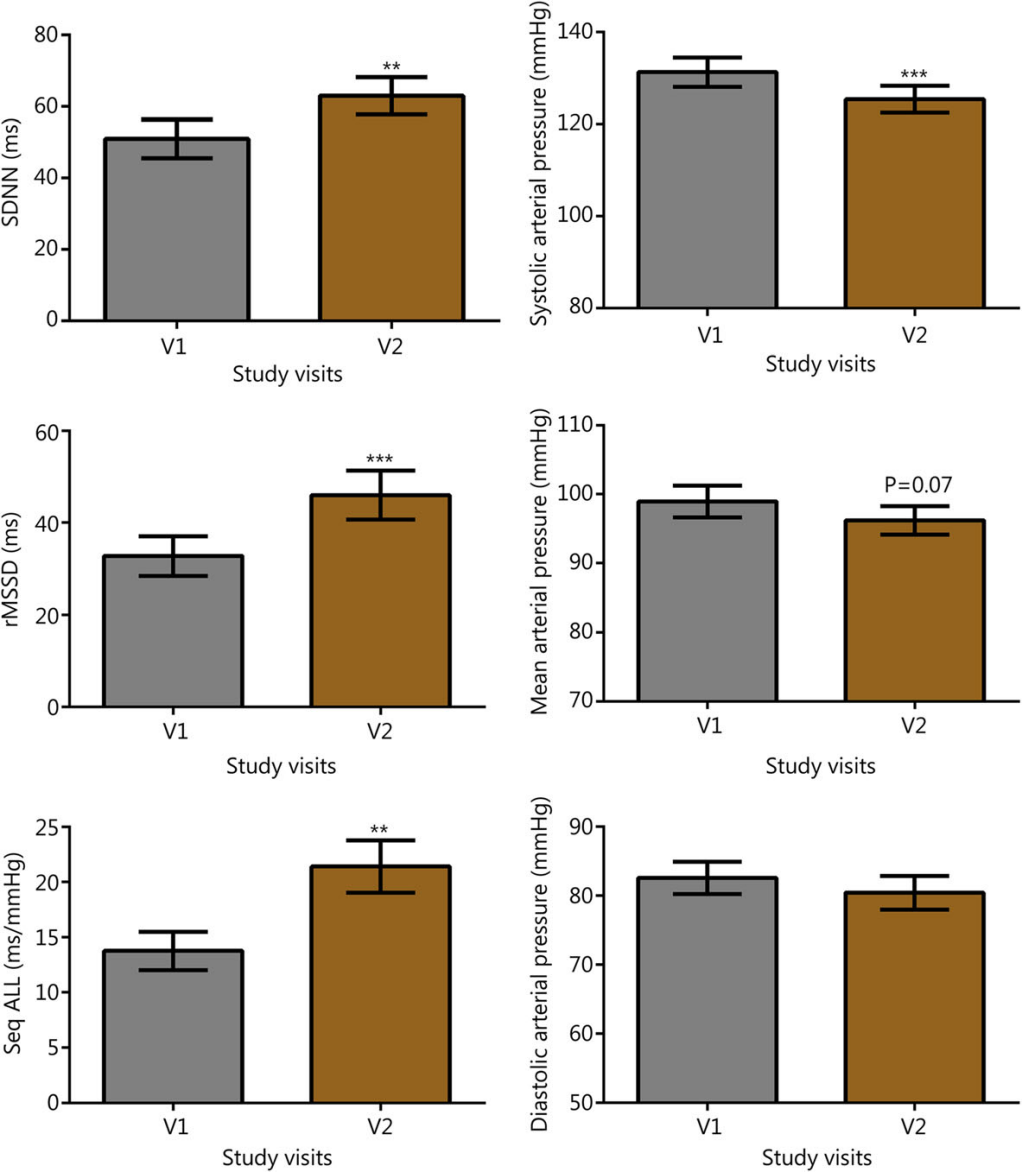
Values for heart rate variability, baroreflex sensitivity, and blood pressure, before and after intervention. Error bars are standard error of the mean (SEM). **, P < 0.01; ***, *P* < 0.001 vs Visit 1 (V1); RRI. R to R interval; SDNN, Standard deviation of the normal beat to normal beat interval; rMSSD, Root mean square of the successive differences; Seq ALL, Sequence all.; V1. baseline study visit; V2. immediately after intervention completion

**Fig. 3:**
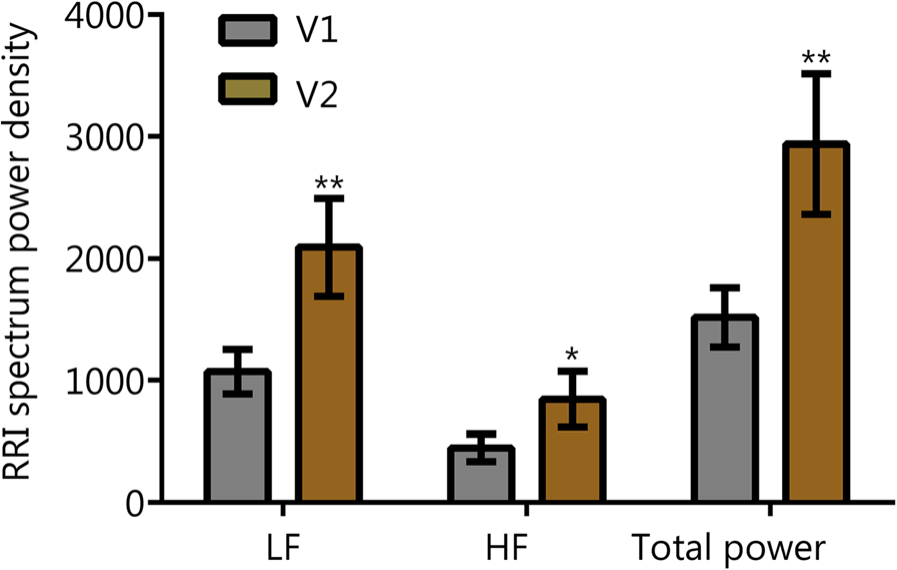
Spectral power values for heart rate variability before and after intervention. Error bars are standard error of the mean (SEM). *, *P* < 0.05; **, P < 0.01 vs V1; RRI, R to R interval; V1, Visit 1; V2, Visit 2; HF, High frequency; LF, Low Frequency. V1. baseline study visit; V2. immediately after intervention completion

**Fig. 4:**
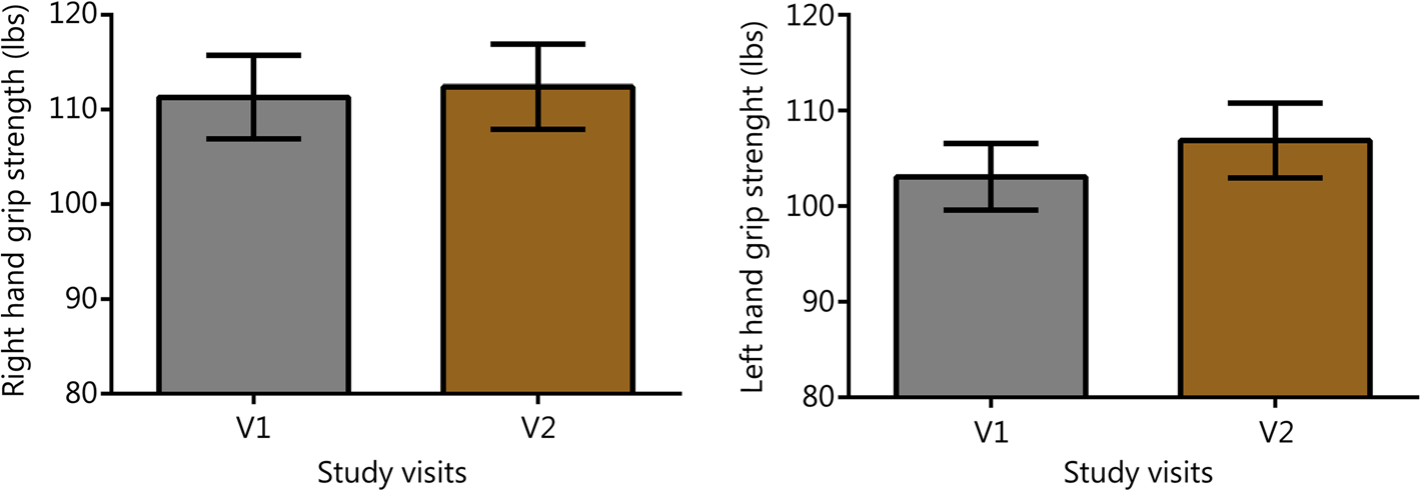
Grip strength before and after intervention, Error bars are standard error of the mean (SEM); V1. baseline study visit; V2. immediately after intervention completion

**Fig. 5:**
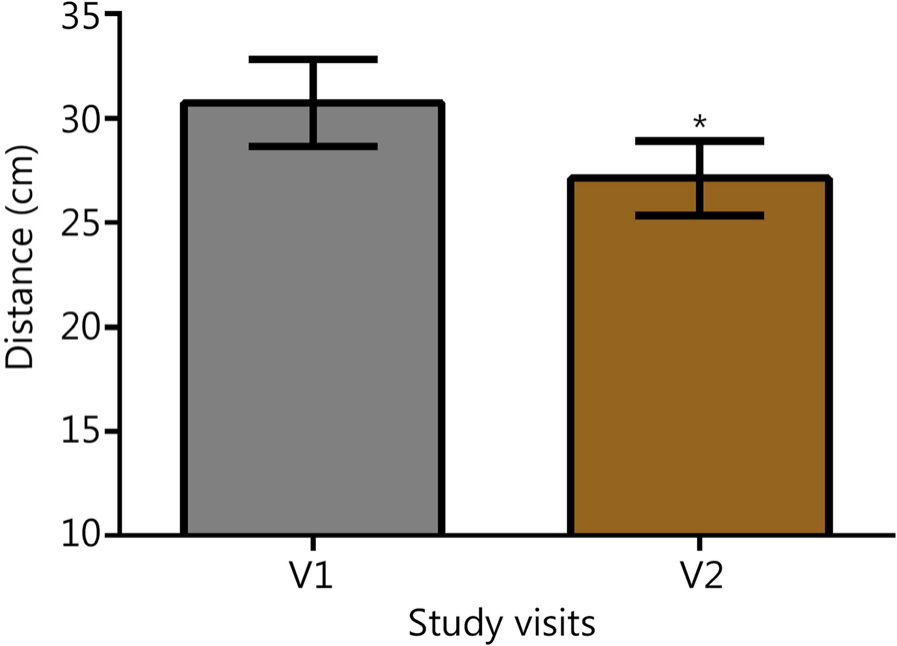
Reaction testing before and after intervention. Error bars are standard error of the mean (SEM). *, *P* < 0.05 vs V1; V1. baseline study visit; V2. immediately after intervention completion

Symptom scores for PTSD, insomnia, depressive mood, and anxiety are shown in Fig. 1. Through the first two follow-up visits, 83% of subjects reported PCL-M scores that were at least 10 points lower than their baseline (at V2, 9 of the subjects reported reductions of at least 10 points on their PCL-M, and at V3, an additional 6 reported reductions of at least 10 points compared to their V1 score). Over the same visits, 78% of subjects reported ISI scores that were at least 7 points lower than their baseline (seven subjects at V2 and an additional 7 subjects at V3). For the 15 individuals with a history of TBI or concussion, there were also durable reductions in concussion-related symptomatology (V2 RPQ -11.8, SD 14.1, *P* < 0.01, subsequent visits not shown). Figures 2 and 3 show V1 and V2 values of HRV, BRS, and blood pressure measures. V1 and V2 values are also shown for grip strength (Fig. 4) and reaction testing (Figure 5). Of the biochemical measures that were assessed, there were trends for reductions in C-reactive protein (−37%, *P* = 0.06), angiotensin II to angiotensin 1–7 ratio (−24%, *P* = 0.19), and IL-10 (−12%, *P* = 0.14). Epigenetic markers showed no statistically significant changes.

## Discussion

This report documents outcomes for a series of active-duty military service members and veterans with symptoms of military-related PTS, predominantly special operations warfighters or support personnel, who participated in the use of a closed-loop, allostatic, acoustic stimulation neurotechnology. On average, there were robust and durable reductions in the symptoms of PTS, insomnia, depressive mood, and anxiety. At the first post-intervention data collection, there were marked increases in HRV and BRS, and there were trends for improvements in physical functional performance and markers of stress or inflammation. There were no adverse events, and all participants completed their course of intervention sessions along with all follow-up data collections.

The present findings are consistent with outcomes reported after the use of closed-loop allostatic neurotechnology by civilians with self-reported PTS, who were mostly women with non-military trauma [45] or athletes with sports-related concussions [46]. Together, these studies concur with the idea that real-time monitoring and modulation of brain activity (closed-loop strategies) can support advanced remediation of neurological and psychiatric disorders, sleep enhancement and, potentially, performance optimization [47–50]. The authors are aware of only two other studies that have reported quantitative HRV effects after the use of any type of intervention by military personnel or veterans with PTSD. HRV decreased with the use of escitalopram [51] and showed no change after the use of mindfulness meditation [52].

Limitations to the generalizability of these findings include the modest sample size and the absence of a control group. Improvements in reaction testing may have been related to practice effects, which have been documented with the drop-stick procedure [53]. The use of numerous types of psychoactive medications, as well as alcohol or recreational drugs, was an exclusion to enrollment, and it is unknown how these co-interventions or influences might affect the outcomes of future studies. Although the improvements demonstrated may have been influenced by subjective expectations, positive social interactions with study personnel, or other “placebo” components, it seems unlikely that these non-specific factors were the fundamental drivers. HRV is an objective physiological measure, and meta-analysis has found that placebo effects in clinical trials tend to be limited to continuous subjective outcomes [54]. In addition, the durability of the symptom score improvements appears inconsistent with the interpretation that changes were due to statistical randomness, regression to the mean, or natural history of disease. Given the protracted duration of symptoms, and numerous other therapies that had been tried previously, spontaneous recovery over a few weeks to months would also be considered unlikely.

Various aspects of the intervention point to its promise as an innovative modality for the remediation of the effects of traumatic stress for active-duty military service members, veterans, and other populations. The reductions in insomnia symptoms are noteworthy given the intractability of sleep complaints in PTSD [55]. Sequelae of TBI can complicate PTSD-specific interventions [56], yet the numerous TBIs reported by the subjects did not appear to hinder their participation, and there was a reduction in TBI-specific symptomatology. The noninvasive methodology is encouraging with respect to safety, feasibility, and scalability considerations. Moreover, support from the US Army Research Office [57] has allowed the development of a self-use configuration of the core technology (Braintellect®-2; Brain State Technologies, Scottsdale, Arizona), with sensor locations at prefrontal and temporal scalp locations only. This device may further facilitate the development of population-based strategies that leverage precision-guided, allostatic neurotechnology, and its standalone use has been proposed as a potential strategy to enable the primary prevention of PTSD through the optimization of sleep quality [58].

## Conclusions

A series of active-duty military personnel and veterans with symptoms of military-related traumatic stress used a closed-loop acoustic stimulation methodology to support the auto-calibration of neural oscillations. Subsequently, participants showed robust improvements in autonomic cardiovascular regulation and durable reductions in PTS-related symptomatology, including insomnia, with no adverse events or dropouts. This study is the first to show an increase in both HRV and BRS, which are significant indicators of the capacity of the brain to exert dynamic and adaptive regulation of peripheral physiology, following an intervention provided to military personnel or veterans with PTS. The composite intervention profile points to the promise of allostatic neurotechnology for system-level PTS management. Ongoing investigations are strongly warranted.

## Abbreviations

Ang II: Angiotensin II
Ang1–7: Angiotensin 1 to 7
BHAM: Bihemispheric autonomic model
BP: Blood pressure
BRS: Baroreflex sensitivity
CES-D: Center for Epidemiologic Studies Depression Scale
CPAP: Continuous positive air pressure
DNA: Deoxyribonucleic acid
DoD: Department of Defense
EMDR: Eye movement desensitization and reprocessing
GAD-7: Generalized Anxiety Disorder 7-item scale
HF: High frequency
HIRREM: High-resolution, relational, resonance-based, electroencephalic mirroring
HR: Heart rate
HRV: Heart rate variability
Hz: Hertz
IL: Interleukin
IRB: Institutional Review Board
ISI: Insomnia Severity Index
LF: Low Frequency
MRI: Magnetic resonance imaging
PCL-M: Posttraumatic Stress Disorder Checklist – Military version
PTS: Posttraumatic stress
PTSD: Posttraumatic stress disorder
rMSSD: Root Mean Square of the Successive Differences
RPQ: Rivermead Post-Concussion Symptoms Questionnaire
RRI: R to R interval
SDNN: Standard deviation of the normal beat to normal beat interval
SNRI: Selective norepinephrine reuptake inhibitor;
SSRI: Selective serotonin reuptake inhibitor
V1–5: Visits 1 through 5
